# Potential Reparative Role of Resident Adult Renal Stem/Progenitor Cells in Acute Kidney Injury

**DOI:** 10.1089/biores.2015.0011

**Published:** 2015-07-01

**Authors:** Fabio Sallustio, Grazia Serino, Francesco Paolo Schena

**Affiliations:** ^1^ Department of Emergency and Organ Transplantation, University of Bari, Bari, Italy.; ^2^ C.A.R.S.O. Consortium, Strada Prov. le Valenzano-Casamassima Km 3, Valenzano, Italy.; ^3^ Dipartimento di Scienze e Tecnologie Biologiche ed Ambientali (DiSTeBA), Università del Salento, Lecce–Monteroni, Lecce, Italy.; ^4^ Schena Foundation, Research Center of Renal Diseases, Bari, Italy.

**Keywords:** renal progenitor cells, acute kidney injury, regenerative medicine, toll-like receptors

## Abstract

Human kidney is particularly susceptible to ischemia and toxins with consequential tubular necrosis and activation of inflammatory processes. This process can lead to the acute renal injury, and even if the kidney has a great capacity for regeneration after tubular damage, in several circumstances, the normal renal repair program may not be sufficient to achieve a successful regeneration. Resident adult renal stem/progenitor cells could participate in this repair process and have the potentiality to enhance the renal regenerative mechanism. This could be achieved both directly, by means of their capacity to differentiate and integrate into the renal tissues, and by means of paracrine factors able to induce or improve the renal repair or regeneration. Recent genetic fate-tracing studies indicated that tubular damage is instead repaired by proliferative duplication of epithelial cells, acquiring a transient progenitor phenotype and by fate-restricted clonal cell progeny emerging from different nephron segments. In this review, we discuss about the properties and the reparative characteristics of high regenerative CD133^+^/CD24^+^ cells, with a view to a future application of these cells for the treatment of acute renal injury.

## Introduction

Acute kidney injury (AKI) remains a major clinical event in nephrology with increasing incidence in both high- and low-income countries.^1^ It represents the sudden loss of the kidney function occurring over a variable range of time (hours/days) and affects up to 7% of hospitalized patients, especially those in medical and surgical intensive care units.^2^ AKI is characterized by acute tubular necrosis that is caused by three main factors: (1) sepsis, (2) nephrotoxicity, and (3) renal ischemia. The pathophysiological events occurring after tubular damage include molecular and cellular mechanisms that have been investigated by several studies,^3–6^ but today the management of AKI is based on conservative therapy such as correction of reversible causes of kidney injury or dialytic treatment. In this review, we will focus on the potential role of resident adult renal stem/progenitor cells (ARPCs) in the regenerative process leading to the repair of tubular damage.

## Cause and Significance of Renal Tubular Injuries

Dysfunction and loss of tubular epithelial cells play a central role in the process underlying the failure of the kidney after ischemic or toxic challenge. The loss of the brush border and the loss of the polarity of the epithelial cell with mislocation of adhesion molecules and Na^+^, K^+^ channel, ATPase, and other proteins are some of the first events in the renal proximal tubular epithelial cells (RPTECs) after the injury. If the insult is severe, cell death by either necrosis or apoptosis occurs.

After detachment from the tubular basement membrane, both injured cells and dead cells can obstruct the tubular lumen, leading to an increase of intratubular pressure that along with backleak of filtrate may contribute to dysfunction.^3–6^ Moreover, some rifts appear in the basement membrane, leading to leakage of molecules from the bloodstream into the tubules. Molecules such as fibronectin and collagen may bind to cells and debris in the lumen and contribute to the obstruction of the tubules.^6^

The kidney has a great capacity for regeneration after tubular damage. The main mechanism includes a principal role of surviving cells that can repair the damaged area, reestablishing the physiological functions, and can repolarize and/or dedifferentiate. Thus, cells migrate to necrotic areas, proliferate, differentiate, and cover the denuded tubular basement membrane.^5,7,8^ The RPTEC regeneration after ischemic or toxic injury could also involve autocrine, paracrine, and/or endocrine growth factors that promote cell proliferation and differentiation.^9–11^ However, the damaged epithelium induces the activation of inflammatory and vasoactive mediators, which can feed back on the vasculature to worsen vasoconstriction and inflammation. In turn, inflammation can contribute in a critical way to expand the injury in AKI.^12^

Nevertheless, in many circumstances, the normal repair program of the kidney may not be sufficient to achieve a successful renal regeneration. In the last years, the source of the cells responsible for the replacement of the injured epithelial tubular cells has been a topic of great interest, especially considering the enthusiasm about the possibility of using adult stem cells therapeutically to improve the regeneration. In fact, in the last few years, many researchers focused their attention on the possible use of stem/progenitor cells to improve regeneration in progressive kidney disease.^13,14^

## Potential Role of Renal Stem/Progenitor Cells in the Repair of Injured Renal Tubules

Recent studies have indicated that adult stem cells could participate in this repair process and in the future might therefore be used in the clinical practice to treat AKI.^15,16^

Adult stem cells are important for self-renewal in tissues, such as the hematopoietic system, the intestine, and the skin, which require a high cell turnover to maintain their homeostasis. Other tissues have a much lower rate of cell turnover, such as the kidney, lung, skeletal muscle, and liver. However, following injury, it has been suggested that repair in these tissues may also involve the recruitment, proliferation, and differentiation of adult stem/progenitor cells.^17^

Many studies have been carried out to explore the possibility of employing adult bone marrow stem cells for this purpose.^18,19^ Hematopoietic stem cells can actively participate in renal regeneration in many animal models of AKI.^6,18,20,21^ In some cases, they support the regeneration of resident cells, but do not participate directly in the repair process.^11,22^

However, other studies, using a genetic fate-tracing approach, excluded the possibility of an extratubular stem or progenitor population migrating into the tubules.^23^

The kidney can completely recover from an ischemic insult and ARPCs could participate in the repair process. Therefore, they might be considered good candidates for future cell-based therapies to improve regeneration in progressive kidney disease. The molecular events that define the regenerative process are supposed to recapitulate nephrogenesis.^24^ In fact, several genes expressed during the embryonic development and downregulated in the mature kidney, such as paired box gene 2 (Pax-2), are reexpressed during the recovery from renal injury.^25^

The ability to regenerate functional tubules after acute injury is an important determinant also in the morbidity of transplanted patients with AKI caused by the delayed graft function (DGF). DGF is a common form of acute renal failure that causes a significant increase in early transplant-related morbidity and a decrease in long-term graft survival complication after cadaveric kidney transplantation. It affects ∼25% of transplant recipients.^26^

Few years ago, we showed the presence of CD133^+^/Pax2^+^ renal progenitor/stem cells in normal kidneys and demonstrated, for the first time, a modulation in the number and proliferating activity of these cells in human transplanted kidneys with acute tubular damage occurring during DGF. As a result of the acute tubular injury in human kidneys, there was a significant increase in progenitor cell number, and most of these cells were proliferating, as demonstrated by the expression of Ki-67. A similar number of CD133^+^/Pax-2^+^ cells was observed in normal kidneys and in pretransplant biopsies of patients with subsequent early graft function. Instead, a significant increase of CD133^+^/Pax-2^+^ cells was found in post-transplant DGF biopsies when compared with their corresponding pretransplant biopsies. These data show an increased activation and proliferation of renal progenitors following tubular damage due to ischemia/reperfusion.^27^

The renal progenitor cells have been identified and isolated by several research groups^28–30^ through the coexpression of CD24 and CD133, two surface molecules that have been used to identify different types of human stem cells,^31–33^ and through the Pax2, a transcription factor that is expressed in the undifferentiated mesenchyme in response to ureteric bud induction and reexpressed in regenerating proximal tubular epithelial cells after acute tubular necrosis.^25^ These cells have a multipotent differentiation ability, comprehending the capacity to differentiate in tubular epithelial cells, osteogenic cells, and adipocytes.^28–30^ CD133^+^ CD24^+^ ARPCs can contribute to the tubular regeneration in mice with glycerol-induced acute renal injury and their administration may ameliorate renal injury and accelerate renal repair.^17^ These cells lack the expression of hematopoietic markers (CD34 and CD45), whereas they express some mesenchymal stem cell (MSC) markers, such as CD29, CD90, CD44, and CD73. They can home to the kidney, integrate into proximal and distal tubules during the repair, and improve the morphology of the damaged renal tissue and the functionality of the kidney.^28,29^

The ARPCs can be isolated both from tubules and glomeruli; they have phenotypical and transcriptional characteristics that are very similar, but with some distinctive differences.^30,34^ CD133^+^CD24^+^ tubular cells (tARPCs) are distinguished from CD133^+^CD24^+^ glomerular cells of the Bowman's capsule (gARPCs) by the CD106 expression; they localize in the proximal tubule and in the connecting segment of tubules. Both tARPCs and gARPCs regenerate tubular cells and improve renal function in SCID mice with AKI and proliferate following injury in the kidney of patients with acute or chronic tubular damage. Moreover, they are both more resistant to injurious agents in comparison with the differentiated renal mature cells.^34^

Recently, a nephron progenitor population was isolated from a human fetal kidney. These cells were positive for the NCAM1 marker and, when cultured *in vitro*, retained their nephrogenic potential and were able to improve the outcome in several kidney injury models.^35^ In addition, NCAM1 downregulated along nephron differentiation can be reactivated in a subset of adult human kidney proximal tubular cells that undergo dedifferentiation to behave as highly clonogenic stem/progenitor cells.^36^ Moreover, metanephric pluripotent stem cells also can generate renal structures as tubules and glomeruli.^37^ Anyway, it is still unclear whether renal function enhancement induced by progenitor cells is due to their differentiation and integration in the injured structures or to their paracrine effect.

## The Debate on the Existence of Renal Stem/Progenitor Cells

Really, despite the several studies carried out on these renal progenitor cells, there is still a debate in the field and the very existence of kidney stem/progenitor cells remains an open question. In fact, some studies, published in the last 2 years, point out different theories. Through a lineage-tracing study, the Humphreys group showed that in mice, subject to injury, there was no dilution of fate marker during the repair step, indicating that unlabeled progenitors do not contribute directly to kidney repair. Moreover, injured proximal tubule epithelia induced expression of markers of putative epithelial stem cells in the human kidney, such as CD24, CD133, and vimentin. These data showed that the principal repair mechanism is due to an injury-induced dedifferentiation of terminally differentiated epithelia.^38^ Similar indications were obtained also by *in vitro* studies showing that human kidney epithelial cells can lose their phenotype, plausibly dedifferentiating, and can adopt a stem cell fate, expressing the CD133 and CD24 markers. They can also generate renal proximal structures upon grafting *in vivo*.^39^ These data make more difficult the interpretations of precursor cells expressing these markers.

Another cell fate-tracking study showed that proximal tubular cells, following different injuries, transiently acquire a high regenerative phenotype with reparative characteristics. Again, their data suggest that a fixed progenitor cell population in the kidney does not exist and, following injury, tubular cells transiently acquire the phenotype of the progenitor cells with reparative characteristics.^40^

In the same year, Dekel's group performed a third genetic lineage-tracing study, differently from the two others, on long-term and unbiased clonal analysis regimen. The authors carried out a clonal analysis of both the developing kidney and kidney during repair and showed a mechanism of continuous cellular renewal of kidney epithelia by fate-restricted and segment-specific clones, beginning from the fetal stage and persisting throughout adult life. In other words, the authors showed that unipotent singly fated clones that continuously maintain and self-preserve the renal mouse kidney tissue throughout life harbor renal progenitor characteristics. These precursors are activated by a WNT signal and, following kidney damage, they regenerate new tubule segments through expansions of single clones that contribute to collective duct or proximal tubules.^41^

## Possible Repairing Mechanisms Driven by ARPCs

Whether the renal progenitors really exist and directly contribute to renal regeneration or do not exist and therefore renal repair originates from dedifferentiated cells or mature cells acquiring a transient progenitor phenotype, the studies on the reparative characteristics of these high regenerative CD133^+^/CD24^+^ cells remain important for potential application in renal medicine and for the development of novel renal regenerative therapies.

**FIG. 1.**
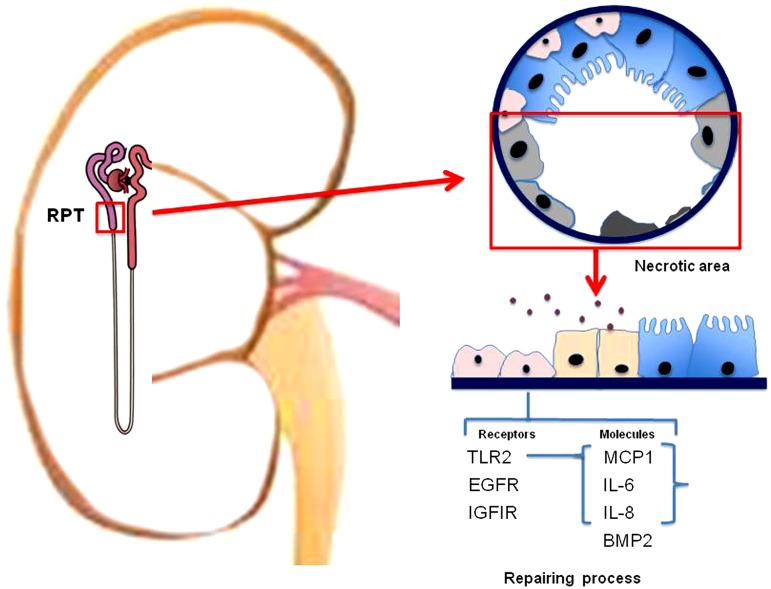
Acute kidney injury causes cell apoptosis and necrosis at the proximal tubular level. Resident ARPCs (pink color) can participate in the repair process both directly, by means of their capacity to differentiate and integrate into the renal tissues, and indirectly, by means of paracrine factors capable to induce regeneration of the surviving tubular epithelial cells (blue color). The ARPCs could detect the injury by TLR2 that recognizes molecules derived from the injured tissue. The TLR2 activation could lead to the secretion of a series of chemokines that stimulate the proliferation of ARPCs themselves to increase the pool of resident cells. Other receptors on ARPCs, such as EGFR and IGF1R, could contribute to the cell activation in the repair process. ARPCs, adult renal stem/progenitor cells; RPT, renal proximal tubule.

A gene expression study of ARPCs by microarrays showed a cluster of genes that discriminated ARPCs from MSCs and RPTECs.^30^ Genes modulated in ARPCs included genes involved in proliferative signal transduction, such as EGFR, IGF1R, and several WNT genes, and immunoresponse activation, such as IL-6, IL-8, and TLR2 (Fig. 1). Among these genes, one of the most interesting is the gene coding for the TLR-2 receptor belonging to the family of toll-like receptors (TLRs). The TLRs recognize the pattern highly conserved at the level of pathogens (PAMPS) that are molecules derived also from injured tissue or necrosis. Therefore, they contribute also to tissue homeostasis. The presence of the TLR-2 has already been demonstrated in MSC^42^ and neural stem cells,^43^ while it was new for the ARPCs. We also showed that renal progenitors secreted MCP-1 and C3 through NF-κB activation in response to TLR2 stimulation. Moreover, TLR2 stimulation by means of specific agonists increased the amount of IL-6 and IL-8 cytokines secreted by ARPCs and increased their proliferation rate.^30^ These molecules could be important in the repair process. In fact, it has been shown that IL-6 stimulates tubular regeneration in rats with glycerol-induced AKI and, through a mechanism of trans-signaling, protects the kidney from further injury.^44,45^ C3, IL-8, and MCP-1 play important roles in modulating stem cell trafficking.^46–48^ In addition, MCP-1 may induce the epithelial-to-mesenchymal transition of RPTECs.^49^ Hence, the TLR2 may function as a sensor of the damage and its activation could lead to the secretion of a series of chemokines useful for the renal repair and could stimulate the proliferation of ARPCs themselves to increase the pool of resident cells and avoid depletion.

Recently, an important role in the renal repair has been assessed for another molecule: the retinoic acid. It promotes differentiation of ARPCs and protects against injury, ameliorating the kidney function in multiple experimental models of AKI. Retinoic acid led to the renal progenitor differentiation into mature podocytes, whereas retinoic acid-induced podocyte differentiation is reduced by proteinuria as a consequence of retinoic acid sequestration by albumin. However, retinoic acid administration can revert renal progenitor differentiation and promote podocyte regeneration.^50,51^

Another mechanism, by which ARPCs can influence the renal repair in some kind of damages, can involve the bone morphogenetic proteins (BMPs). BMPs are particularly important in stem cell biology because they regulate the stem cell differentiation and function in different tissues^52–54^ and are reexpressed in the adult kidney following renal injury,^55^ particularly in regenerating proximal tubular cells.^56^ Specifically, the BMP-2 is mainly expressed *in vivo* in tubular and glomerular ARPCs and a dramatic increase in BMP-2 occurs in ARPCs following renal DGF.^57^

It can also induce epithelial-to-mesenchymal transition^58,59^ and myofibroblastic differentiation, increasing levels of α-SMA, collagen I, and fibronectin in ARPCs, but not in proximal tubular epithelial cells. In graft biopsies of patients with DGF, a marked increase of α-SMA expression in CD133^+^ cells was observed confirming the myofibroblastic transition of ARPCs.^57^

Even though fibrosis is a part of the normal pathophysiological response to injury in many tissues, the excessive healing and the excess of collagen and other matrix components at sites of chronic inflammation can lead to scar tissue formation and progressive tissue injury.^60^ Therefore, it should be taken into consideration that ARPCs could have a positive or negative role in reverse renal fibrosis depending on the type and time of exposure to injuries (chronic infections, toxic and metabolic injuries, idiopathic inflammatory diseases). Indeed, progenitor cells are responsive to a fine regulation system in which BMP-2 mediates a negative feedback loop, balancing differentiation and proliferation by means of opposing effects on cell induction or proliferation.^26,57,61^

There is one more mechanism that ARPCs could use to induce regeneration of damaged tissues: the secretion of microvesicles (MVs). Several studies revealed that adult stem cells can influence the phenotype of injured cells by transferring proteins, bioactive lipids, mRNA, and microRNAs in the MVs and altering the fate of the target cells. MVs released from stem/progenitor cells may confer a stem cell-like phenotype to injured cells, with the consequent induction of self-regenerative programs.^62–64^ In fact, it has been shown that MVs derived from endothelial progenitor cells can protect the kidney from ischemia–reperfusion injury by delivering miRNAs and reprogramming the resident renal cells.^65,66^ Furthermore, the treatment with MVs derived from MSCs enhanced the morphological and functional recovery of glycerol-induced AKI in SCID mice.^67^ Further studies on MVs derived specifically from CD133^+^/CD24^+^ cells are needed to understand whether this repairing mechanism is used also by ARPCs and what molecules are involved.

## The ARPCs: Versatile and Fascinating Cells

Another interesting finding about the renal progenitors is that aquaporins (AQP) 1, 3, and 5 are expressed at both mRNA and protein levels in ARPCs. This observation led to the discovery of the AQP5 presence in the mammalian kidney, whose expression was previously unknown.^68^ In fact, AQP5, a water channel having a prominent expression in salivary glands and the lung,^69–71^ was also expressed both in human ARPCs and in kidneys of mouse, rat, and humans. The expression is, however, weak and restricted to the apical membrane of pendrin-positive type-B intercalated cells in the connecting tubule and cortical collecting duct. The AQP1 and AQP3 are expressed in the proximal tubule and collecting duct, respectively,^72^ suggesting that tARPCs exhibit molecular features of epithelial cells from different portions of the kidney tubule. However, the role of AQP5 is not so clear and raises questions concerning its function in the kidney. It has been shown that AQP5 can have a role in human cell proliferation and migration in different tissues and cancer cells^73–75^; therefore, we can speculate that in renal cells also or at least in ARPCs it could have a similar role.

The possibility that ARPCs could be used to enhance the repair of renal injury, directly or indirectly through their paracrine factors, is really exciting, but potential risks derived from their use should be taken into account. Side effects as tumor formation, proangiogenic properties, and inflammatory consequences should be investigated deeply. In effect, some recent studies reported a CD133^+^ cell population found in human clear cell carcinomas, the most widespread renal cancer. These cells exhibited most part of markers of renal progenitors derived from healthy renal tissue, such as Pax2, CD24, and CD73, and had the same mesenchymal phenotype and differentiative capacity of their normal counterpart.^76,77^ Instead, these tumoral CD133^+^ renal cells were not able to form tumors in immunodeficient mice, even if they can enhance the growth and vascularization of the tumor when cotransplanted with renal tumor cells.^76^

On the other hand, recently, a further putative cancer stem cell marker that distinguishes the tumoral CD133^+^ renal cells from the normal ARPCs has been identified. This marker, the CTR2, is a membrane marker involved in renal cell carcinoma-derived cell cisplatin resistance. These CD133^+^ CTR2^+^ tumoral cells are clonogenic, express embryonic stem cell markers, and are tumorigenic *in vitro* and angiogenic *in vivo*.^77^

Therefore, the importance of better understanding the behavior of renal CD133^+^ stem/progenitor cells and their physiological and pathological responses appears clear. Recently, a microengineered biochip resembling the structure of a kidney proximal tubule that embeds ARPCs has been reported.^78^ The device allowed recovery of urea, creatinine, and glucose of 20%, 13%, and 52%, respectively. Moreover, the exposition of ARPCs to a fluid shear stress in the chip induced AQP2 to localize at the apical region of the cells and the Na^+^, K^+^, ATPase pump at the basolateral portion of cells, indicating a well-organized cell polarization, in contrast to statically cultured ARPCs. This renal microdevice could be used to further investigate ARPCs *in vitro* or to test drugs for their toxic effects.

In conclusion, considering these experimental studies all together, we can realize that ARPCs have a remarkable regenerative potential for therapeutic purpose in AKI. They could be exploited in a future therapeutic protocol in different ways that are not antithetical to each other, but can be complementary. The ARPCs could be administrated after having oriented or increased their activity *ex vivo*; alternatively, they could be first conditioned in an environment resultant from a specific renal damage, and then their regenerative paracrine factors could be isolated and administered. Further studies, both *in vitro* and *in vivo*, are needed to deepen our knowledge on these versatile, potentially effective, and fascinating ARPCs and to make closer the possibility to use them to repair renal injury.

## Abbreviations Used

AKI: acute kidney injury
AQP: aquaporins
ARPCs: adult renal stem/progenitor cells
BMPs: bone morphogenetic proteins
DGF: delayed graft function
MSC: mesenchymal stem cell
MVs: microvesicles
Pax-2: paired box gene 2
RPTECs: renal proximal tubular epithelial cells
TLRs: toll-like receptors
